# The Treatment of Primary Lymphoepithelioma‐Like Carcinoma in the Head and Neck and Nasopharyngeal Carcinoma

**DOI:** 10.1002/cam4.70389

**Published:** 2024-11-14

**Authors:** Qiaohong Lin, Mingyuan Du, Shida Yan, Xing Zhang, Xiyuan Li, Ying Zhang, Shiting Zhang, Shuwei Chen, Ming Song

**Affiliations:** ^1^ State Key Laboratory of Oncology in South China, Guangdong Key Laboratory of Nasopharyngeal Carcinoma Diagnosis and Therapy, Guangdong Provincial Clinical Research Center for Cancer Sun Yat‐Sen University Cancer Center Guangzhou China; ^2^ Department of Head and Neck Surgery Sun Yat‐Sen University Cancer Center Guangzhou China

**Keywords:** EBV infection, Lymphoepithelioma‐like carcinoma, nasopharyngeal carcinoma, prognosis, radiotherapy, surgery

## Abstract

**Background:**

An uncommon cancer, lymphoepithelioma‐like carcinoma (LELC) resembles undifferentiated nasopharyngeal carcinoma (NPC) histologically. The aim is mainly to introduce the diagnosis and treatment of LELC and compare it with NPC in our descriptive study.

**Methods:**

A total of 278 patients with NPC and 157 patients with head and neck LELC had their medical records examined in this study. The propensity score matching (PSM) approach was employed to attain a 1:1 match between the LELC and NPC groups. Kaplan–Meier analysis was performed for overall survival (OS) of LELC and NPC. To determine their predictive values for OS, univariate and multivariate Cox regression analyses with significant survival differences (*p* < 0.05) were carried out.

**Results:**

Similar to NPC, 107 (68.2%) LELC cases had Epstein–Barr virus (EBV) infection. LELC of the parotid glands was present in nearly 46.5% of patients with head and neck LELC. Most patients were treated with surgery with neck dissection. After PSM, LELC had similar 5‐year OS rates to NPC (81.6% vs. 79.0%). LELC was less prone to distant metastasis compared to NPC. Age, T stage, N stage, and distant metastases were found to be substantially correlated with the outcome of LELC, according to the multivariate Cox regression analysis (*p* < 0.05).

**Conclusions:**

EBV infection in the head and neck has been associated with LELC and NPC. When compared to NPC, LELC is more likely to arise in the salivary glands and has a lower incidence of distant metastasis. Surgery with neck dissection is the primary treatment for LELC.

## Graphical abstract

Both LELC and NPC are associated with EBV infection in the head and neck. Compared with NPC, LELC is more likely to occur in the salivary glands and has a lower incidence of distant metastasis. The main treatment for LELC is surgery with neck dissection.

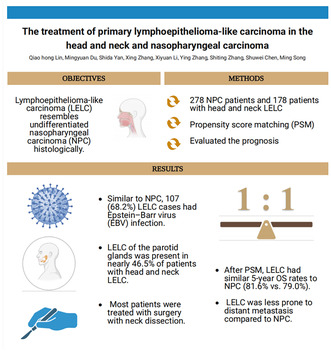

## 1 Introduction

Lymphoepithelioma‐like carcinoma (LELC) is a rare solid malignancy and was first described in the nasopharynx by Regaud and Schminke in 1921 [1, 2]. LELC is characterized as a poorly differentiated carcinoma with extensive lymphocytic infiltration and is commonly found in head and neck [3]. Based on surveillance, epidemiology, and end results (SEER) database, the total incidence of LELC is 0.091/100,000 person‐years and over 75% of cases were located in the head and neck, including nasopharynx and other nonnasopharyngeal region [4]. It is believed that head and neck LELC is linked to Epstein–Barr virus (EBV) infection and those located at nasopharynx were further defined as nasopharyngeal carcinoma (NPC) [5, 6]. The southern China and Southeast Asia, the areas with the highest incidence of EBV, account for more than 80% of the cases of NPC [7, 8]. However, unlike NPC, the incidence of LELC in the head and neck was relatively low and associated studies were mainly case reports and small‐scale analyses [9, 10].

The diagnosis of LELC of head and neck is tricky due to the similarity between NPC and LELC. Once diagnosed as LELC, the metastasis of NPC must be excluded by histopathological biopsy and imaging examination. Moreover, the treatment options are distinctly different between two diseases. The widespread application of intensity‐modulated radiotherapy and chemotherapy had contributed to improved survival with reduced toxicities for patients with NPC [11]. However, surgery is still the main course of therapy for patients with head and neck LELC, while radiotherapy and chemotherapy are less sensitive for them [12, 13]. Due to the scarce incidence and public unawareness, current advances in therapeutic development of LELC in the head and neck were limited.

Therefore, we believe that it is necessary to make comparisons between LELC and NPC and investigate the clinical characteristics, prognosis and therapy of LELC in the head and neck. In this study, we collected a single‐center retrospective analysis on patients with LELC in the head and neck in a large‐scale sample size and compared them with NPC patients using propensity score matching (PSM).

## 2 Methods

### 2.1 Patients and Variables

This study examined patients who were diagnosed with head and neck LELC between the years 2000 and 2018, and patients diagnosed with NPC from 2008 to 2012 at Sun Yat‐sen University Cancer Center. This work was authorized by the Sun Yat‐sen University Cancer Center Ethics Review Committee.

Inclusion criteria were as follows: (1) histopathology confirmation of primary LELC or NPC; (2) age of patients ≥ 18 years; (3) the completion of follow‐up and clinical data. The medical records regarding demographic parameters, laboratory tests, pathological features, and treatment strategies were simultaneously retrieved. Nasopharyngeal carcinomas are diagnosed as NPC according to histopathological morphology of nasopharyngeal biopsy. In patients with LELC, biopsy of the local mass was performed to diagnose LELC according to histopathological morphology. Meanwhile, nasopharyngeal biopsy or imaging examination should be performed to exclude NPC. Patients with a history of NPC were also excluded from LELC cohort.

For LELC patients, the primary method of detecting EBV infection was in situ hybridization (ISH) of Epstein–Barr early RNA (EBER), and EBV DNA copy number in the real‐time quantitative PCR (EBV DNA ≥ 1000 copies) for NPC patients. The clinical stage of all cases was re‐evaluated by sophisticated physicians and radiologist using the 8th edition of the American Joint Committee on Cancer staging criteria (8th AJCC‐TNM staging system). T0 was defined as no primary tumor found. In patients with clinical N1–N3, therapeutic neck dissection was performed with cervical lymph nodes. In patients with clinical N0, elective neck dissection was performed with cervical lymph nodes. NLR was defined as the ratio of neutrophils to lymphocytes before treatment.

Patients who received radiotherapy were treated with intensity‐modulated radiotherapy (IMRT) or three‐dimensional conformal radiotherapy (3D‐CRT). The time from initial diagnosis to death or last follow‐up was called overall survival (OS).

### 2.2 Statistical Analysis

All statistical analyses are performed using IBM SPSS Statistics 25.0 and Graphpad Prism 8.0.2. Variables are summaries of numbers and percentages. To reduce selection bias and adjust for different characteristics of patients, propensity score matching (PSM) was used for 1:1 matching between LELC and NPC groups with a caliper of 0.05. Gender, age, NLR and TNM stage were the matched factors.

To determine prognostic differences in LELC and NPC between subgroups, Kaplan–Meier survival analysis and log‐rank test were used, and variables with significant survival differences were used to perform multivariable Cox regression analyses to determine their predictive values for OS of LELC in the head and neck. Statistically significant was defined as a two‐tailed test with a *p* value < 0.05.

## 3 Results

### 3.1 Clinical Characteristics

The study included 157 LELC patients and 278 NPC patients. Table 1 shows the baseline clinical information of these patients. The LELC patients had a median age of 44 years (range 18–76 years), and the NPC patients had a median age of 45 years (range 18–74 years). One hundred and seven (68.2%) LELC patients and 196 (70.5%) NPC patients were infected with EBV.

**TABLE 1:** Baseline characteristics of LELC and NPC patients before and after PSM.

Variables	Before PSM			After PSM		
	LELC (%)	NPC (%)	*p*	LELC (%)	NPC (%)	*p*
Gender			0.002			0.302
Male	88 (56.1)	196 (70.5)		88 (56.1)	97 (61.8)	
Female	69 (43.9)	82 (29.5)		69 (43.9)	60 (38.2)	
Age (years)			0.008			0.531
≤ 50	92 (58.6)	173 (62.2)		103 (65.6)	98 (62.4)	
> 50	65 (41.4)	105 (37.8)		54 (34.4)	59 (37.6)	
NLR			0.369			0.495
≤ 2.605	120 (76.4)	174 (62.6)		91 (58.0)	85 (54.1)	
> 2.605	37 (23.6)	104 (37.4)		66 (42.0)	72 (45.9)	
EBV status						< 0.001
Negative	6 (3.8)	82 (29.5)		6 (3.8)	48 (30.6)	
Positive	107 (68.2)	196 (70.5)		107 (68.2)	109 (69.4)	
Unknown	44 (28.0)	—		44 (28.0)	—	
T stage			< 0.001			< 0.001
T0‐2	115 (73.2)	67 (24.1)		115 (73.2)	36 (22.9)	
T3‐4	42 (26.8)	211 (75.9)		42 (26.8)	121 (77.1)	
N stage			< 0.001			< 0.001
N0‐1	81 (51.6)	231 (83.1)		81 (51.6)	129 (82.8)	
N2‐3	76 (48.4)	47 (16.9)		76 (48.4)	28 (17.8)	
*M* stage			< 0.001			0.013
M0	152 (96.8)	261 (93.9)		152 (96.8)	140 (89.2)	
M1	5 (3.2)	17 (6.1)		5 (3.2)	17 (10.8)	
TNM stage			0.034			0.107
I‐II	42 (26.8)	50 (18.0)		42 (26.8)	30 (19.1)	
III‐IV	115 (73.2)	228 (82.0)		115 (73.2)	127 (80.9)	
Surgery						
No	21 (13.4)	278 (100.0)		21 (13.4)	—	
Tumor resection	41 (26.1)	—		41 (26.1)	—	
Tumor resection and neck dissection	95 (60.5)	—		95 (60.5)	—	
Radiotherapy			< 0.001			< 0.001
No	62 (39.5)	7 (2.5)		62 (39.5)	7 (4.5)	
Yes	95 (60.5)	271 (97.5)		95 (60.5)	150 (95.5)	
Chemotherapy			< 0.001			< 0.001
No	97 (61.8)	21 (7.6)		97 (61.8)	10 (6.4)	
Yes	60 (38.2)	257 (92.4)		60 (38.2)	147 (93.6)	

Abbreviations: EBV, Epstein–Barr virus; NLR, neutrophils/lymphocytes; TNM, the 8th AJCC‐TNM staging system.

The origin of the LELC in the head and neck is summarized in Table 2. Ninety‐eight (62.4%) patients had LELC originated from the salivary glands, and almost 46.5% of patients had LELC from the parotid glands, 14 (8.9%) patients received a diagnosis of LELC along with a primary location unknown for cervical lymph node metastases.

**TABLE 2:** Survival by subsite of LELC in the head and neck after PSM.

Tumor site	Number (%)	5‐year OS (%)	10‐year OS (%)
Salivary gland	98 (62.4)	84.3	72.8
Nasal cavity and paranasal sinus	14 (8.9)	70.1	70.1
Oral cavity	11 (7.0)	81.8	81.8
Oropharynx	13 (8.3)	75.5	75.5
Orbital cavity	7 (4.5)	66.7	44.4
Neck mass	14 (8.9)	85.7	58.8
LELC	157 (100)	81.6	70.6
NPC	157 (100)	79.0	72.3

Abbreviations: LELC, lymphoepithelioma‐like carcinoma; NPC, nasopharyngeal carcinoma; OS, overall survival.

Due to the tumors having no obvious symptoms or painless masses, 115 (73.2%) LELC patients and 228 (82.0%) NPC patients were late stage (stage III–IV) before PSM. Five (3.2%) of the LELC patients had distant metastases. Two (1.3%) patients had liver metastases and the remaining patients had brain, lung, or bone metastases. Among NPC patients with distant metastases, 13 (4.7%) patients had bone metastases, which was the most common site of metastasis.

### 3.2 Treatments

LELC patients in our cohort received multidisciplinary therapies. A total of 136 (86.6%) individuals underwent surgery, 95 (60.5%) of whom had cervical lymph node dissections (24 for elective neck dissections and 71 for therapeutic neck dissections). Radiation doses ranging from 54 to 74 Gy were given in 30–32 portions to the tumor area or neck for 55 (35.0%) patients who got postoperative radiotherapy and 36 (22.9%) patients who received postoperative chemoradiotherapy. Fourteen (8.9%) nonsurgical patients receiving chemoradiation or radiotherapy alone received 54–74 Gy, 25–33 fractions. Sixty (38.2%) patients received chemotherapy, which was based on platinum‐based drugs. Among LELC patients with distant metastases, two (40.0%) accepted chemotherapy, one (20.0%) surgery and adjuvant radiotherapy and one (20.0%) discontinued. One (20.0%) patient with surgery and adjuvant chemoradiotherapy survived to the end of the follow‐up.

Different from LELC, the main treatment for NPC is radiotherapy, 271 (97.5%) patients in our study accepted radiotherapy. Meanwhile, 257 (92.4%) of the patients also received platinum‐based regimens of chemotherapy, of which 17 (6.1%) patients received only induced chemotherapy without concurrent radiotherapy. In the distant metastatic cohort, 10 (58.8%) patients chose chemoradiotherapy, and seven (41.2%) patients had systemic chemotherapy.

### 3.3 Survival Analysis for OS

**FIGURE 1:**
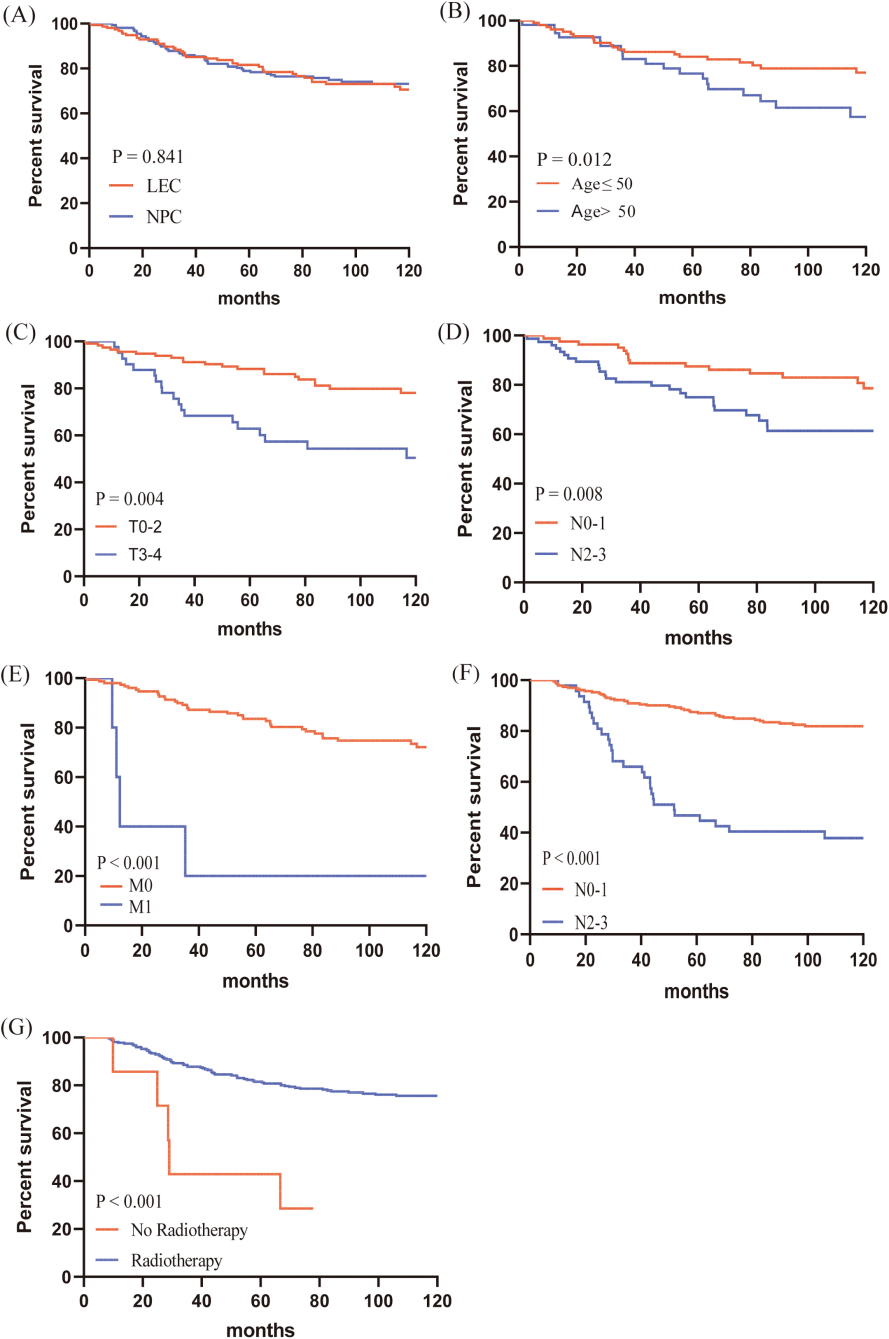
Kaplan—Meier survival analyses to estimate OS for LELC in the head and neck (A) and NPC (A) patients after PSM. Kaplan—Meier survival analyses to estimate OS for LELC in the head and neck by age (B), T stage (C), N stage (D), and M stage (E). Kaplan—Meier survival analyses to estimate OS for NPC in the head and neck by N stage (F) and radiotherapy (G) in the whole set.

After PSM, there were 157 patients in each group. The follow‐up date was censored on December 31, 2022, the median follow‐up period was 106.07 months (IQR: 64.40 months–144.33 months). After finishing following up, 43 (27.4%) LELC patients and 46 (29.3%) NPC patients had died. According to the results of the Kaplan–Meier survival analyses, we found that patients in the LELC group had a comparable 5‐year (81.6% vs. 79.0%, *p* > 0.05, Figure 1A) and 10‐year (70.6% vs. 72.3%, *p* > 0.05, Figure 1A) OS rate to those with NPC.

For the LELC cohort, univariate Cox regression analysis demonstrated that individuals aged 50 years old or younger (*p* < 0.005) or those with stage T0–T2 (*p* < 0.001) exhibited a more favorable prognosis, and the patients with stage N2–N3 (*p* = 0.008), M1 (*p* < 0.001), or late stage (stage III–IV, *p* = 0.02) had significantly worse OS. Furthermore, multivariate Cox analysis showed that in patients with head and neck LELC, the following significant prognostic factors were found: age (*p* = 0.012, Figure 1B), T stage (*p* = 0.004, Figure 1C), N stage (*p* = 0.008, Figure 1D), and M stage (*p* < 0.001, Figure 1E) (Table 3).

**TABLE 3:** Significant factors associated with OS of LELC patients on univariate analysis and multivariate analysis by Cox regression.

Variables	Univariate analysis			Multivariate analysis		
	HR	95% CI	*p*	HR	95% CI	*p*
Age (years)			0.027			0.033
≤ 50	1			1		
> 50	1.972	1.082–3.594		1.928	1.056–3.522	
Gender			0.894			
Male	1					
Female	0.96	0.526–1.753				
NLR			0.091			
≤ 2.605	1					
> 2.61	1.735	0.917–3.285				
EBV status			0.311			
Negative	1					
Positive	0.473	0.111–2.012				
T stage			< 0.001			0.002
T0‐2	1			1		
T3‐4	2.872	1.574–5.240		2.564	1.397–4.709	
N stage			0.008			0.006
N0‐1	1			1		
N2‐3	2.327	1.250–4.332		2.511	1.300–4.849	
M stage			< 0.001			< 0.001
M0	1			1		
M1	7.68	2.706–21.794		10.736	3.461–33.299	
TNM stage			0.02			
I‐II	1					
III‐IV	2.8	1.179–6.650				
Surgery			0.221			
No	1					
Tumor resection	0.422	0.158–1.128				
Tumor resection and neck dissection	0.599	0.271–1.323				
Radiotherapy			0.559			
No	1					
Yes	0.836	0.458–1.526				
Chemotherapy			0.49			
No	1					
Yes	0.794	0.413–1.527				

Abbreviations: EBV, Epstein–Barr virus; NLR, neutrophils/lymphocytes; TNM, the 8th AJCC‐TNM staging system.

In addition, the treatment regimens differed among primary tumor sites (Table 2). Eight (72.7%) patients with oral cavity LELC main had surgery combined with other treatments. However, for LELC of the orbital cavity, they mainly chose surgery alone, even abandoning surgery altogether. Hence, different treatment regimens contributed to the difference in survival. The best 10‐year OS of LELC arising in the oral cavity was 81.8%, and the worst 10‐year OS of LELC arising in the orbital cavity was 44.4%.

In the NPC patients enrolled, favorable prognostic factor was identified for patients without lymph node metastasis (*p* < 0.001, Figure 1F) or with radiotherapy (*p* < 0.001, Figure 1G) following univariate and multivariate Cox regression analysis (Table 4).

**TABLE 4:** Significant factors associated with OS of NPC patients on univariate analysis and multivariate analysis by Cox regression.

Variables	Univariate analysis			Multivariate analysis		
	HR	95% CI	*p*	HR	95% CI	*p*
Age (years)			0.287			
≤ 50	1					
> 50	1.285	0.809–2.041				
Gender			0.42			
Male	1					
Female	1.235	0.740–2.060				
NLR			0.019			
≤ 2.605	1					
> 2.605	1.72	1.093–2.705				
EBV status			0.013			
Negative	1					
Positive	2.087	1.167–3.733				
T stage			0.172			
T0‐2	1					
T3‐4	1.5	0.839–2.683				
N stage			< 0.001			< 0.001
N0‐1	1			1		
N2‐3	4.425	2.769–7.072		5	2.837–7.266	
M stage			0.001			
M0	1					
M1	3.252	1.607–6.580				
TNM stage			0.01			
I‐II	1					
III‐IV	2.773	1.273–6.044				
Radiotherapy			< 0.001			< 0.001
No	1			1		
Yes	0.195	0.078–0.489		0.174	0.069–0.438	
Chemotherapy			0.084			
No	1					
Yes	3.457	0.848–14.088				

Abbreviations: EBV, Epstein–Barr virus; NLR, neutrophils/lymphocytes; TNM. the 8th AJCC‐TNM staging system.

In the distant metastasis cohort, four (80.0%) LELC patients and nine (52.9%) NPC patients died. In LELC patients with distant metastases, survival ranged from 9.57 to 130.97 months, and only one patient still alive who was underwent surgery and adjuvant chemoradiotherapy. LELC patients had a shorter 5‐year OS than NPC patients (20.0% vs. 52.9%, *p* = 0.118), but this was not a statistically significant difference.

## 4 Discussion

In the study, we presented a cohort of 157 patients with LELC and 278 patients with NPC. Compared to NPC, LELC in the head and neck was also highly associated with EBV infection. Meanwhile, LELC is more likely to arise in the salivary glands and the majority of patients were locally advanced stages when diagnosed. Unlike radiotherapy for NPC, radical resection with neck dissection was the primary treatment for LELC. The 5‐year and 10‐year OS for LELC were 81.6% and 70.6%, respectively, which were similar to the prognosis of NPC after PSM. Age, T stage, N stage, and distant metastases were found to be substantially correlated with the outcome of LELC.

Notably, LELC and NPC share an adjacent anatomical location in the head and neck, and are commonly found in southern China [14]. As a high‐incidence area of NPC, it is necessary to differentiate it with LELC in the head and neck. LELC is uncommon in the head and neck; therefore, little is known about its clinical behavior or how it responds to therapy. Prior research has either been conducted on a small scale or through database analysis. It is necessary to comprehend how to distinguish LELC in the head and neck from NPC and the treatment between them.

In the LELC cohort, as previous studies have shown, the salivary gland was the most frequently damaged site (almost 80%), and the parotid gland was 46.5% in our study [12, 15]. Nonetheless, the oropharynx was the most often affected site of LELC in both Europe and the United States [14]. In our study, only 8.3% of patients occurred in the oropharynx.

The carcinogenesis of LELC is still unclear. Similar to NPC, there is a strong association between EBV infection and LELC in head and neck. Interestingly, despite the differences in clinical presentation and anatomical site, there are common features that EBV drive immune‐suppressed tumor immune microenvironment primarily in viral‐driven cancers and similar in somatic mutational landscape, which also demonstrate how the transforming virus affects epithelial cells [5]. Since southern China has a higher rate of EBV infection than northern China, there is a correlation between the two regions' differences in LELC incidence [13, 15]. Ma et al. argued that EBV infection is closely associated with LELC and may be a causative factor for LELC, especially in areas with high incidence of EBV, like Alaska, Greenland, and Southeast Asia [5, 6, 14]. With regard to the low incidence of EBV, for example, the Caucasian population has a tendency to have EBV‐negative LELC [14].

Regarded as the “gold standard”, EBER in situ hybridization is used to detect EBV in tumor cells. In our cohort, of the 157 LELC patients, 113 underwent EBER in situ hybridization. Excluding undetected patients, our cumulative results showed that 94.7% of the enrolled LELC patients were infected with EBV, which coincided with the clinical characteristics of LELC in the endemic region [16, 17]. However, as in other studies, EBV infection did not affect the prognosis of LELC patients in our study (*p* = 0.311) [12, 14].

NPC is sensitive to radiotherapy. As the primary treatment for NPC patients, radiotherapy could improve the prognosis of NPC (*p* < 0.001). Regarding treatments for LELC of the head and neck, there remains disagreement. In our study, LELC patients with radiotherapy did not have a better prognosis (*p* = 0.559). Thirty‐nine out of 69 patients in a retrospective analysis by Ma et al., got postoperative radiation [14]. Although the relapse‐free survival (RFS) of these individuals was higher, their overall survival (OS) did not significantly improve [14]. 75.9% of patients in a study by Lin and colleagues received radiotherapy, nevertheless, there was no discernible difference in the outcomes for LELC treated with or without radiotherapy (77% vs. 74%, *p* = 0.33) [4]. In the subgroup analysis, radiotherapy significantly improves cancer‐specific survival (CSS) compared with no radiotherapy group for NPC, but not for LELC [4]. From the point of view of somatic cell molecule's mutational landscape, NPC have more abundant chromosomal instability, global hypermethylation, and histone modification, which were less frequently observed in LELC [5]. This partly explains why NPC are more sensitive to radiotherapy. Notably, patients with extremely dangerous recurrence reasons following surgery, which include R1 resection, by microscopy positive profit margins or lymph node metastases, were occasionally advised to get radiation [14].

Without a doubt, the first option for patients with head and neck LELC is surgery, even for those diagnosed at advanced stages [13, 18]. 86.6% of LELC patients in our study were accepted with surgery or in conjunction with additional therapies. Compared to patients who did not undergo surgery (75.6%), the 5‐year OS of LELC treated with surgery is 82.6%. However, the survival analysis showed no significant survival benefit (*p* = 0.119), which is consistent with previous studies [13, 18]. There were 63.7% of lymph node metastasis cases in our study, which was comparable to previous studies with an incidence rate of 40%–80% [14]. When there was clinical suspicion of lymph node metastases (stage N1–N3), patients without neck dissection had a 5‐year OS of 76.2%, whereas those with it was 81.8% (*p* = 0.867), but was not statistically different.

Few research has been done on head and neck LELC distant metastases. In an investigation of 179 salivary gland LELC, two patients (1.11%) had distant metastasis [19]. In the research by Petruzzi et al., distant metastases were present in two out of 41 individuals with laryngeal and hypopharyngeal LELC [20]. Among the enrolled individuals with head and neck LELC, five patients had distant metastasis and the median OS was 39.79 months. The only patients who were still alive underwent surgery and adjuvant chemoradiotherapy. The results of the study were that comprehensive treatment may be an option for patients with distant metastases. In endemic NPC, the incidence of distant metastases ranges from 6% to 8% at the time of presentation [21]. 10.8% of patients had distant metastasis in our study. They mainly accepted chemotherapy‐based treatment, and the median OS was 63.7 months. Patients with LELC with distant metastases have a worse prognosis than those with NPC (*p* = 0.118), but there was no significant difference. Due to its rarity, we need more cases to find a suitable treatment to improve the prognosis of LELC patients with distant metastases.

Our research has various limitations. Initially, our selection of patients might be subject to selection bias due to the scarcity of LELC. Second, because there are no medical records, the recurrence of LELC cannot be acquired. Therefore, we are unable to identify the patterns of recurrence and look into the variables linked to disease‐free survival. Third, it is necessary to conduct multicentered studies to obtain external validation of the results obtained.

In conclusion, LELC is a rare neoplasm, and is similar to NPC in the head and neck. They are both associated with EBV infection. When compared to NPC, LELC is more likely to arise in the salivary glands and has a lower incidence of distant metastasis. Surgery with neck dissection is the primary treatment for LELC.

## Author Contributions

**Qiaohong Lin:** data curation (equal), formal analysis (equal), methodology (equal), writing – original draft (lead). **Mingyuan Du:** conceptualization (equal), supervision (equal), validation (lead), writing – original draft (equal). **Shida Yan:** conceptualization (equal), resources (equal), supervision (lead). **Xing Zhang:** data curation (equal), formal analysis (equal), software (equal), supervision (equal). **Xiyuan Li:** investigation (equal), validation (equal). **Ying Zhang:** investigation (equal), visualization (equal). **Shiting Zhang:** methodology (equal). **Shuwei Chen:** funding acquisition (equal), writing – review and editing (lead). **Ming Song:** conceptualization (lead), funding acquisition (lead), methodology (lead), writing – review and editing (equal).

## Ethics Statement

The Institutional Review Board of our university approved the current retrospective study (No. B2022‐193‐01), and the ethics committee waived the requirement for informed consent.

## Consent

Since this is a retrospective study employing biological data and medical records from past clinical practices, patient consent was not required.

## Conflicts of Interest

The authors declare no conflicts of interest.

## Data Availability

The data presented in this study are available on request from the corresponding author.
