# Correction to “The Treatment of Primary Lymphoepithelioma‐Like Carcinoma in the Head and Neck and Nasopharyngeal Carcinoma”

**DOI:** 10.1002/cam4.70751

**Published:** 2025-03-13

Lin Q, Du M, Yan S, Zhang X, Li X, Zhang Y, Zhang S, Chen S, Song M. The Treatment of Primary Lymphoepithelioma‐Like Carcinoma in the Head and Neck and Nasopharyngeal Carcinoma. *Cancer Med*. 2024 Nov;13(22):e70389. doi: 10.1002/cam4.70389

The article category has been changed from “Review” to “Research Article.”

We apologize for this error.

